# Wear Mechanisms and Notch Formation of Whisker-Reinforced Alumina and Sialon Ceramic Tools during High-Speed Turning of Inconel 718

**DOI:** 10.3390/ma15113860

**Published:** 2022-05-28

**Authors:** Chao Xue, Dong Wang, Jingjing Zhang

**Affiliations:** School of Mechatronic Engineering, Xi’an Technological University, Xi’an 710021, China; wangdong@xatu.edu.cn (D.W.); zhangjingjing@xatu.edu.cn (J.Z.)

**Keywords:** nickel-based alloys, ceramic tools, wear mechanisms, notch formation

## Abstract

Nickel-based alloys, referred to as the most difficult-to-cut materials, pose a great challenge to cutting tool materials due to their excellent high-temperature properties. Ceramic tools have the potential to improve the machinability of these alloys with the advance of toughening mechanisms. In this work, the wear mechanisms of SiC whisker-reinforced alumina and Sialon when high-speed turning Inconel 718 alloy under dry cutting condition were investigated. The results showed that the wear process of Al_2_O_3_-SiC_w_ WG300 was dominated by the notch wear, while the flank wear characterized by ridges and grooves perpendicular to the cutting edge was the main wear mode for Sialon SX9. A Ti−enriched belt was found at the boundary of the wear band for both ceramic tools. The SEM inspection and EDS analysis for this belt suggested the trace of diffusion between the workpiece material and tool matrix. As for the notch formation, the periodically adhesive action of the workpiece material at the depth-of-cut line combined with the thermal shock resistance of ceramic tools were considered to account for its formation. In addition, the oxidation of the workpiece material at the depth-of-cut line played a positive role in reducing the adhesive affinity and consequent notch wear.

## 1. Introduction

Nickel-based alloys are widely applied in the manufacture of key components and structures for aerospace, nuclear, marine, and petrochemical industries [1]. Specifically, these alloys become indispensable to the hot sections of aero-engines such as compressor discs and combustion chambers of gas turbines. Nickel-based alloys such as Inconel, Nimonic, Udimet, and Rene are successfully manufactured in wrought, cast, or sintered form to provide excellent high-temperature strength and good corrosion and oxidation resistance [2]. These alloys are always referred to as the most difficult-to-cut materials. Among them, Inconel 718 is the most frequently available grade. The superior properties ensure the service performance of components in aggressive environments but pose a serious challenge to cutting tool materials. During machining of nickel-based alloys, extremely high cutting forces and temperature at the cutting zone always lead to short tool life and unfavorable surface integrity, due to the excellent high-temperature strength, low thermal conductivity, strong work hardening tendency, and high chemical affinity for tool materials of these alloys [3].

The poor machinability of nickel-based alloys confines cutting speeds to low levels when machining with cemented carbide tools. Cantero [4] carried out dry and lubricated finish turning of Inconel 718 under cutting speeds of 50 m/min and 70 m/min. The TiAlN/TiN coated carbide tools suffered from notch wear, flank wear, and chipping in the tests. Polvorosa [5] compared the machinability of Inconel 718 and Waspaloy using uncoated carbide tools at different coolant pressures. It was found that the flank wear was lower when turning Waspaloy compared to Inconel 718, and notch wear became a controlling factor in cases with large grain size. Ezugwu [6] reported that the triple-layer TiN/TiCN/TiN coated tools outperformed the single-layer TiAlN coated tools when machining Nimonic C-263 at a higher cutting speed of 68 m/min. It was the strong tendency to burr formation of Nimonic C-263 and high temperature gradient that undermined tool performance, particularly for the single-layer TiAlN coated tools. Although many high performance PVD or CVD coatings are successfully applied for carbide tools, these tools are not suitable for high-speed machining of nickel-based alloys because they cannot withstand the extremely high cutting forces and temperature at the cutting zone.

Ceramic cutting tool materials have gained increasing application in high-speed machining of nickel-based alloys with the advance of various toughening mechanisms. Whisker-reinforced alumina (Al_2_O_3_-SiC_w_) and Sialon possessing enhanced fracture toughness and thermal shock resistance are considered as the most suitable materials for high-speed machining of these alloys. The fracture toughness and tensile strength of Al_2_O_3_-SiC_w_ are enhanced by the strong silicon carbide whiskers randomly dispersed throughout the brittle alumina matrix [1]. The fracture process of the matrix is retarded, and total failure will occur as the whiskers are pulled out from the matrix. The matrix also has an improved thermal conductivity due to the incorporation of the whiskers. Silicon nitride (Si_3_N_4_) ceramic is recognized as one of the toughest ceramic materials. Sialon was developed from Si_3_N_4_ ceramic with alumina and yttria (Y_2_O_3_) as the sintering aids [7]. Sialon, having low coefficient of thermal expansion and high thermal conductivity, is also recommended for use in high-speed machining of nickel-based alloys.

Li [8] and Fernández-Lucio [9] compared the cutting performance of Sialon ceramic and coated carbide tools during high-speed machining of Inconel 718. Cutting speeds of 300 m/min in turning and 680 m/min in milling could be achieved with Sialon. The high cutting speeds and consequent high material removal rates were much higher than those achieved with coated carbide tools. Although the high-performance ceramic tools impart high efficiency to the manufacture of nickel-based alloys, the potential of these ceramic tools is severely restricted by notch wear at the depth-of-cut line, which may lead to catastrophic failure of tools. The notch formation and wear mechanisms of ceramic cutting tools were investigated by various researchers. Narutaki [10] reported that SiC whisker-reinforced alumina showed good performance with respect to notch wear in the cutting speed range of 100–300 m/min. The notch wear was considered to be caused by adhesion of workpiece material to the tool, while the flank wear was generated by the diffusion between workpiece material and ceramic tool elements. Sun [11] also suggested that the flank wear was mainly caused by diffusion and chemical wear during high-speed milling of GH4099 with Sialon ceramic tools. In addition, the chemical reaction between Sialon and GH4099 also led to notch wear and failure of the tool. Ezugwu [12] attributed the notching to the strain hardening of the workpiece during machining. The ceramic tool was continuously rubbed by the work-hardened chip, and thus tool particles could be removed by abrasion and/or attrition wear mechanisms. Zhuang [13] also reported that the work-hardened layer beneath the machined surface was the main cause of notch wear, and further proposed a notch wear prediction model based on the hardened layer depth and the notch geometry for Sialon ceramic tool. Lee [14] supported that the notching was a consequence of irregular separation of the edge of the chip from the workpiece. This separation was largely considered as a tearing process, which favored interrupted seizure and consequent pullout of tool material. The effect of the adhered workpiece material on tool wear was also reported by Shalaby [15]. The Si-O tribo-film was found on the worn cutting edge, which indicated that the oxidation happened to the SiC whiskers. The investigations of Wayne [16] and Zhao [17] reported that a severe temperature gradient could be produced at the depth-of-cut line during machining. This thermal shock played an important role in the notch formation of ceramic tools.

To improve the performance of ceramic cutting tools when machining nickel-based alloys, hybrid machining techniques were successfully developed by some researchers. Shin [18] conducted plasma enhanced machining (PEM) experiments of nickel-based alloy Inconel 718. Results showed that the notch wear of SiC whisker-reinforced alumina tool could be suppressed, thereby resulting in longer tool life. Obikawa [19] investigated air jet assisted (AJA) machining of Inconel 718 at high cutting speeds. Experiment results revealed that the notch wear was no longer the dominant wear mode of SiC whisker-reinforced alumina tool under the combined action of an air jet applied to the tool tip and coolant. This change was attributed to the formation of tribo-film through the tribo-chemical reaction between SiC and water, which was activated by oxygen supplied by the air jet.

Although the notch wear could be reduced and consequent longer tool life was obtained with the introduction of hybrid machining techniques, no consensus has yet been reached as to the formation mechanism of notches. In addition, wear mechanisms determining performance of ceramic cutting tools are still complicated under high-speed cutting conditions, especially in terms of the diffusion process between workpiece material and cutting tool elements. The purpose of the present research was to study the wear mechanisms and notch formation of ceramic cutting tools by comparing the wear performance of SiC whisker-reinforced alumina and Sialon in high-speed turning of Inconel 718 alloy. A preliminary cutting test was first conducted to identify the proper cutting parameters for the tool wear experiments. Then, the wear behaviors of ceramic tools under different cutting speeds were evaluated. Further, the wear mechanisms behind wear behaviors were identified and the explanation for the notch wear was proposed. This research contributes to the further understanding of the wear mechanisms of ceramic tools during machining of nickel-based alloys. It could provide guidance for the design and manufacture of new ceramic cutting tools, especially ceramic tools coated with innovative coating materials for high-efficiency machining of nickel-based alloys.

## 2. Experimental Details

The workpiece material used in the present study was solution-treated and aged nickel-based alloy Inconel 718. The turning experiments were carried out on an Inconel 718 bar with dimensions of 140 mm in diameter and 250 mm in length. The chemical composition, mechanical, and physical properties of the workpiece material are listed in Table 1 and Table 2, respectively. A 45° chamfer was prepared on the tested bar prior to each cutting entry to reduce potential impact on the cutting edge of ceramic inserts.

Two types of commercially available ceramic tools recommended for machine nickel-based alloys were used in the experiments: the SiC whisker-reinforced alumina WG300 from Greenleaf and the Sialon SX9 from NTK. All ceramic inserts were square-shaped (ISO designation SNGN 120416T01020) and were clamped in a CSRNR 2020K12 type tool holder to offer a rake angle of −6°, a clearance angle of 6°, an inclination angle of −6°, and a cutting edge angle of 75°.

All the cutting experiments were conducted on an EMCO-Maxxturn 65 turning center with a maximum spindle speed of 5000 r/min under dry cutting condition. The Al_2_O_3_-SiC_w_ is recommended by cutting tool manufactures to be applied at higher cutting speeds, while the Sialon suitable for higher feed rates cutting conditions. Thus, a preliminary cutting test was conducted to identify the proper cutting parameters for the tool wear experiments.

According to ISO 3685: 1993, the tool wear criteria adopted for the cutting experiments were the average flank wear VB ≥ 0.3 mm, notch wear VN ≥ 0.6 mm, severe chipping, or fracture of the cutting edge. Cutting experiments were stopped when any one of the criteria was reached. A Dino-Lite ^TM^ digital camera with an image processing software was used for tool wear measurement, and a Hitachi S-530 scanning electron microscope (SEM) coupled with an Oxford Link-ISIS energy dispersive X-ray spectroscope (EDS) was applied to the observation and analysis of the worn surfaces.

## 3. Results and Discussion

### 3.1. Preliminary Cutting Test

**Figure 1:**
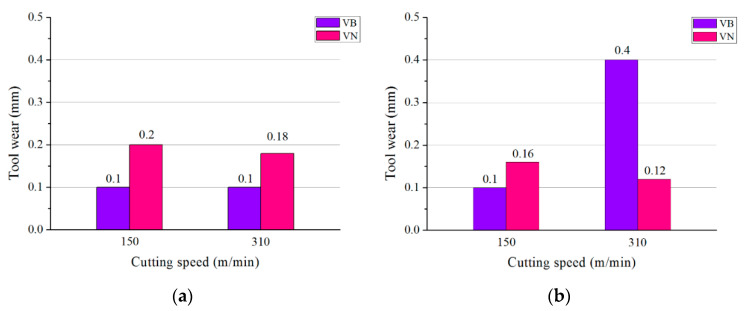
Tool wear of ceramic tools after 30 s of cutting: (**a**) WG300; (**b**) SX9.

To identify the proper cutting parameters applied in the tool wear experiments, a 30 s short-time cutting test was carried out at a cutting speed of 150 m/min and 310 m/min. The feed rate of 0.1 mm/r and the depth of cut of 1 mm were held constant in the two tests. The average flank wear VB and notch wear VN of WG300 and SX9 recorded after the cutting test are shown in Figure 1. At the cutting speed of 150 m/min, the average flank wear VB of WG300 and SX9 both exceeded 0.1 mm. A V-shaped groove, which is the typical characteristic of notch wear, was found to appear at the depth-of-cut line for both ceramic tools. Although the notch wear VN of WG300 attained 0.2 mm which was slightly larger than 1.6 mm of SX9, the notching area of WG300 was much wider. When the cutting speed came to 310 m/min, the wear modes of the two ceramic tools became quite different. The VB of SX9 was 0.4 mm and already attained the tool wear criteria, while WG300 had no evident change in the value of VB. Additionally, slight notching was generated for SX9 as compared to WG300, and the VN of the two ceramic tools both decreased with the increase in cutting speed. The results of the short-time cutting test showed that the cutting speed had a great effect on the wear of ceramic tools and Al_2_O_3_-SiC_w_ and Sialon should be employed in different cutting speed ranges. In the present research, the effect of cutting speed on tool wear was emphasized, and the feed rate and the depth of cut were held constant for both ceramic tools. The cutting parameters applied in the tool wear experiments are listed in Table 3.

### 3.2. Wear Processes

**Figure 2:**
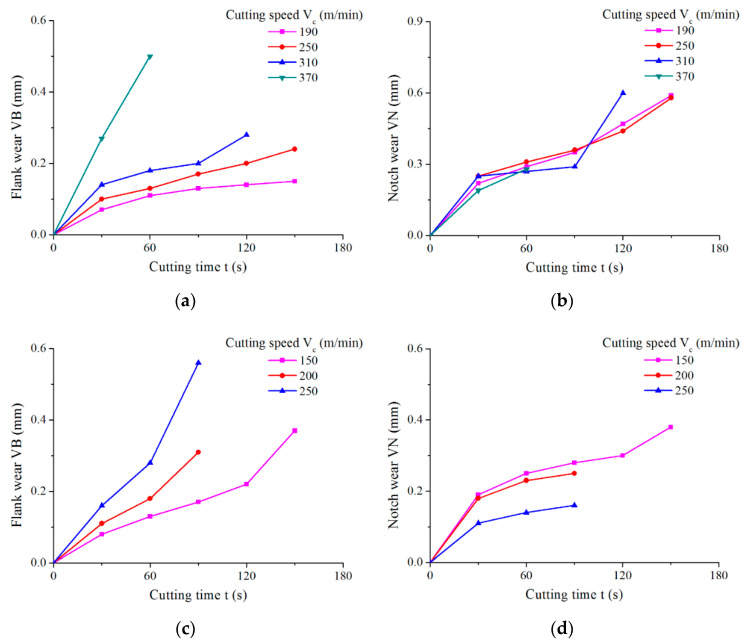
Tool wear curves at different cutting speeds: (**a**) VB of WG300; (**b**) VN of WG300; (**c**) VB of SX9; (**d**) VN of SX9.

Based on the results of the preliminary cutting test and the recommendations from cutting tool manufactures, higher cutting speeds were arranged for WG300, while higher feed rates for SX9 in the tool wear experiments (Table 3). Wear behaviors of the two ceramic tools under different cutting speeds were evaluated in terms of the average flank wear VB and notch wear VN. The tool wear measurement was performed at each 30 s cutting interval. The wear processes of WG300 and SX9 are illustrated in Figure 2. As seen from Figure 2, with increase in cutting speed, the VB of the two ceramic tools both increased, but the VN of the two ceramic tools presented an opposite trend, especially for SX9. The failure mode of WG300 was notch wear, which dominated the whole tool wear process at low cutting speeds (Figure 2b). However, when the cutting speed came to 370 m/min, the failure mode changed to the flank wear (Figure 2a), and the tool attained the wear criteria with severe nose wear. As for SX9, the dominant failure mode was flank wear at all cutting speeds (Figure 2c). In addition, the notch wear decreased gradually as the cutting speed increased from 150 m/min to 250 m/min (Figure 2d).

### 3.3. Wear Patterns

**Figure 3:**
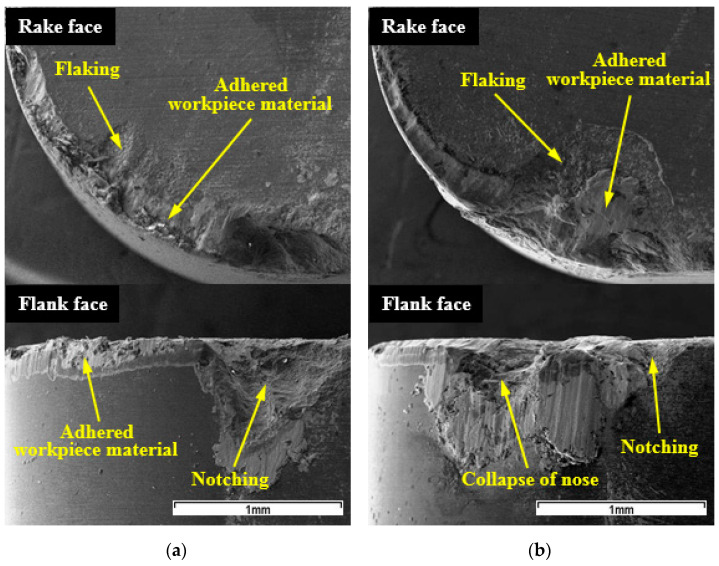
SEM micrographs of wear morphologies of WG300 at: (**a**) 190 m/min; (**b**) 370 m/min.

The wear morphologies of WG300 obtained from SEM observation are shown in Figure 3. The worn flank face was characterized by adhered workpiece material and notching at all cutting speeds (Figure 3a), while the rake face was found to be susceptible to flaking. As the cutting speed increased, the edge chipping and flaking of the rake face caused by brittle fracture became more severe (Figure 3b). Additionally, notching at the minor cutting edge and the collapse of the tool nose could be also detected at high cutting speeds. Similar to the wear morphology of coated carbide tool in turning Inconel 718 [20], the adhesion of the workpiece material onto the worn faces was prominent, which was considered to play an important role in the tool wear process.

**Figure 4:**
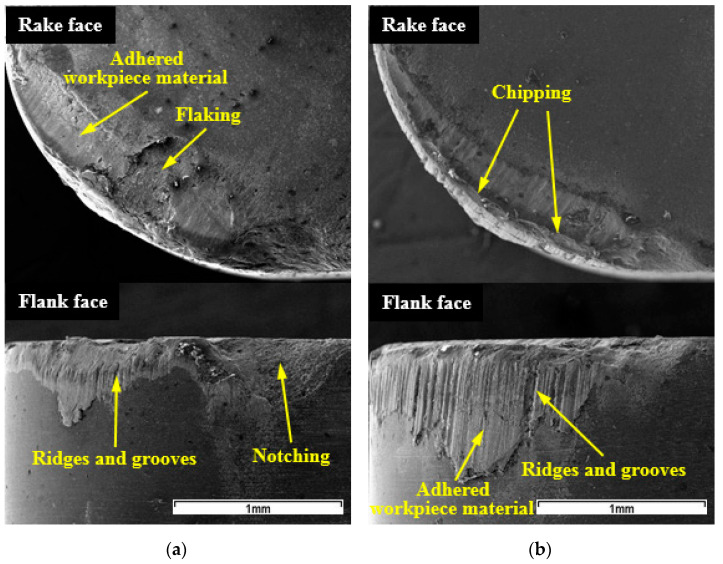
SEM micrographs of wear morphologies of SX9 at: (**a**) 150 m/min; (**b**) 250 m/min.

Compared with WG300, adhesion of workpiece material onto the worn faces was relatively uniform for SX9. As seen from SEM micrographs of SX9 (Figure 4), apparent ridges and grooves perpendicular to the cutting edge were generated on the flank face. The notching was also found at the depth-of-cut line, and it regressed with an increase in cutting speed. At a cutting speed of 250 m/min, the notching nearly disappeared and severe ridges and grooves covered whole worn flank face (Figure 4b). Similar to WG300, SX9 also suffered from edge chipping and flaking of the rake face.

### 3.4. Wear Mechanisms

#### 3.4.1. Abrasion

**Figure 5:**
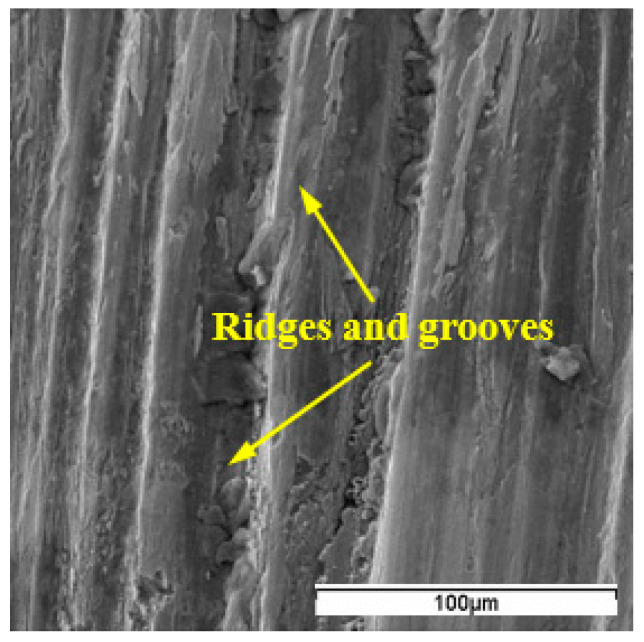
Ridges and grooves on the flank face of SX9.

Ridges and grooves along the chip flow and workpiece travel directions are referred to as the typical characteristic of abrasive wear [21]. These ridges and grooves were generated on the flank face under all cutting conditions for SX9. Typical ridges and grooves obtained at a cutting speed of 250 m/min are shown in Figure 5. The SEM observation illustrated that the flank wear of SX9 was chiefly caused by abrasion. As for WG300, no apparent ridges and grooves were found due to the adhesion of the workpiece material. However, at the boundary of the wear band, tiny grooves could still be detected. The abrasive wear was deemed to be caused by the scraping action of hard carbide particles such as NbC and TiC contained in the Inconel 718 alloy [22]. Although ceramic tools are hard and can retain their hardness at high temperatures, fragments of tool material were still scraped away when these hard particles moved over the tool face in high frequency, thus leading to grooves. Moreover, through SEM inspection it was found that the range of chipping corresponded to that of grooves. Tool particles would drop from the tool matrix when the tool experienced chipping or flaking. The particles sandwiched between the tool and workpiece also resulted in grooves with chip flow or workpiece travel.

#### 3.4.2. Adhesion

**Figure 6:**
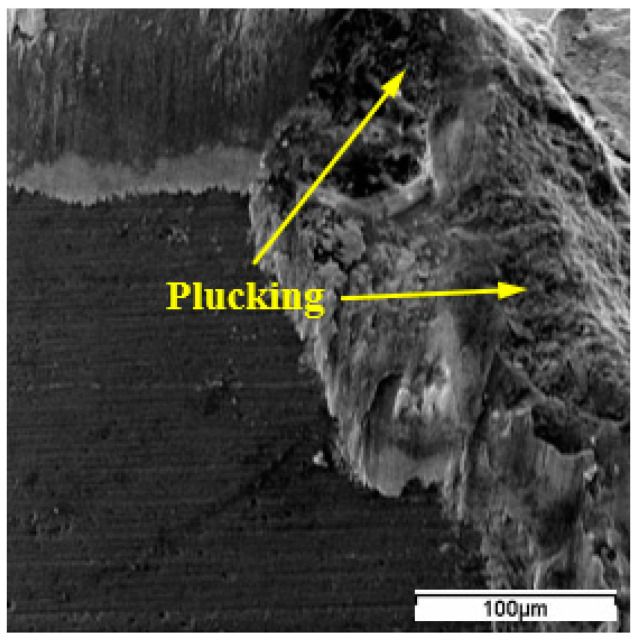
Plucking at the notching area of WG300.

Adhesion of the workpiece material onto the worn faces was found to be prominent in the tool wear experiments. The adhesive wear was believed to be related to the uneven flow of the workpiece material under conditions of seizure [23]. Periodic adhesion of the workpiece material onto the tool faces is a prerequisite to the adhesive wear. Flaking and plucking readily happened to the ceramic cutting tools under the tearing action of the workpiece material due to their relatively low shear strength and tensile strength. Plucking could be detected at the notching area and on the flank face for WG300 (Figure 6). Concaves were left on the worn face after tool material was torn off from the tool matrix with chip flow or workpiece travel. Flaking on the rake face of WG300 and SX9 was also the result of adhesive wear.

#### 3.4.3. Diffusion and Chemical Reactions

**Figure 7:**
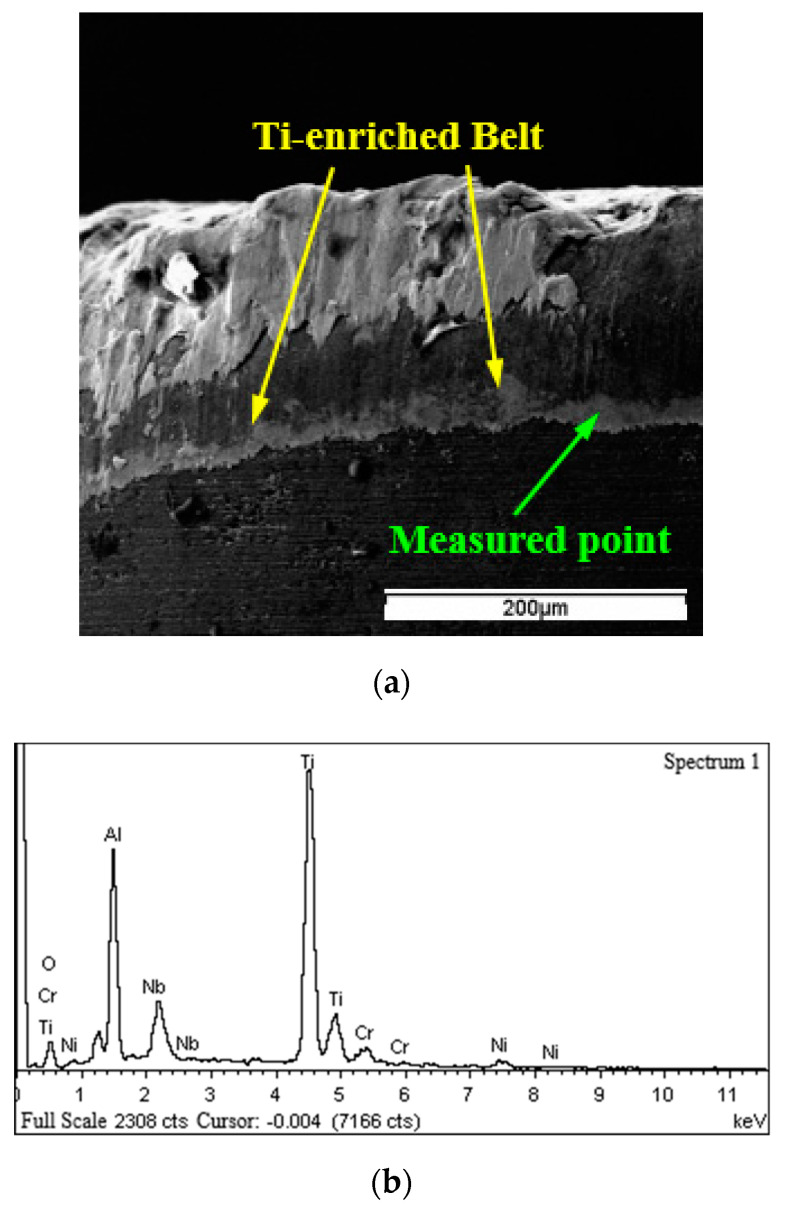
Ti−enriched belt on the rake face of WG300: (**a**) SEM micrograph; (**b**) EDS spectrum.

**Figure 8:**
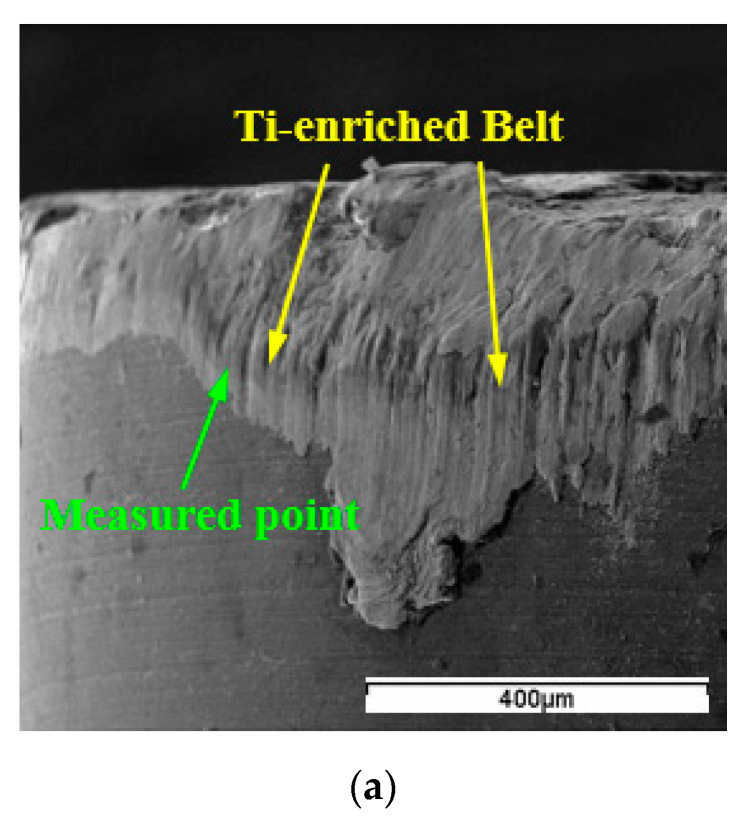

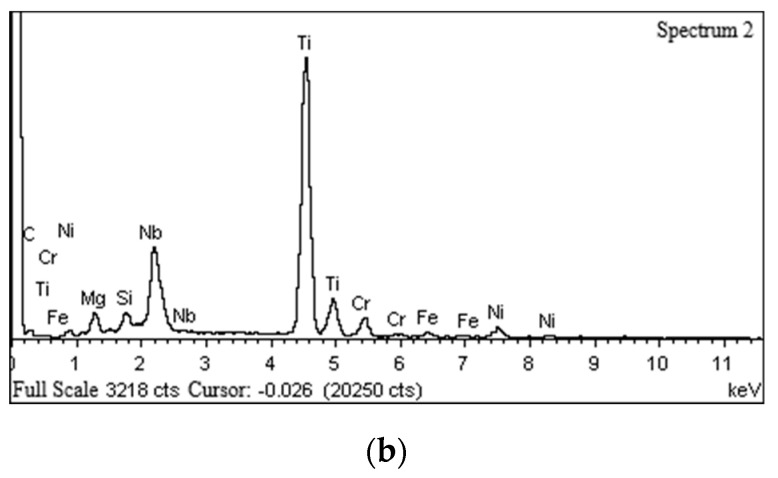
Ti−enriched belt on the rake face of SX9: (**a**) SEM micrograph; (**b**) EDS spectrum.

Besides SEM inspection of the tool faces, EDS analysis of elements was also conducted at wear regions of WG300 and SX9. The results of SEM inspection and EDS analysis showed that a Ti−enriched belt was generated under all cutting conditions applied in the experiments. As seen from the wear band of WG300 obtained at a cutting speed of 190 m/min (Figure 7a), a belt appeared at the boundary of the wear band on the flank face. The result of the corresponding EDS analysis demonstrated that the element composition of this belt differed from that of the adhered workpiece material. As shown in Figure 7b, the content of element Ti attained a high level, whereas the content of elements Ni, Fe, and Cr greatly decreased compared to their original contents in the Inconel 718 alloy. As for SX9, the Ti−enriched belt was not only detected at the boundary of the wear band (Figure 8a), but also at the boundary of the crater on the rake face. The results of corresponding EDS analysis demonstrated a similar variation in the content of element Ti, as shown in Figure 8b.

When turning Inconel 718 with ceramic tools at high cutting speeds, the cutting temperature measured on the rake and flank faces both reached 1200 °C [24]. Such high cutting temperature generated at the cutting zone could facilitate the diffusion process between workpiece material and cutting tool elements. Brandt [25] observed that elements Ni, Fe, and Cr penetrated along the silicon nitride grain boundaries into the matrix of the Sialon tool, while Ti and Nb were left on the tool surface and formed a (Ti, Nb)-nitride coating. Bushlya [26] also found the diffusion of elements Ni, Fe, and Cr into the tool matrix when turning Inconel 718 with the SiC whisker-reinforced alumina tool. These elements dissolved the SiC whiskers by replacing the silicon in the whiskers.

In the present study, the phase constitution analysis of Ti was not conducted to identify the chemical state of the Ti−enriched belt, but the EDS analysis results proved that the enrichment of Ti on the tool surface was related to the diffusion between workpiece material and cutting tool elements. The elements Ni, Fe, and Cr from Inconel 718 could diffuse along the SiC whiskers or silicon nitride grain boundaries into the tool matrix, whereas Ti was enriched on the tool surface due to immediate reaction with elements from the cutting tools. The SiC whiskers or silicon nitride grains were dissolved by the elements diffused into the tool matrix, and thus the tool matrix was undermined. Individual grains of WG300 and SX9 would be removed from the matrix under the action of abrasion or adhesion mechanism, resulting in the wear of the ceramic tools. The Ti−enriched coating could not hold a stable state on the tool surface because of the scraping action of the chip or workpiece. At the main section of the chip/tool or workpiece/tool contact area, the Ti−enriched coating was scraped away from the tool surface due to high shearing stress. However, at the boundary of the contact area, the coating that withstood lower shearing stress was left in the form of belt. The stable state of the Ti−enriched coating might also be broken by periodic attachment and detachment of adhered workpiece material.

### 3.5. Notch Formation

A V-shaped notching considered as the typical wear characteristic of ceramic tools was generated at the depth-of-cut line for both WG300 and SX9. If the notching was mainly caused by chemical reactions between the ceramic tools and workpiece material, the notch wear would deteriorate at higher cutting speeds because the chemical reactions readily occurred at high temperatures generated under high cutting speed conditions. In addition, the Al_2_O_3_-based ceramic tools with high chemical stability would be superior to Si_3_N_4_-based tools in resisting the notch wear. However, the two ceramic tools exhibited quite a distinct resistance to the notch wear. The notch wear nearly dominated the whole wear process of WG300, but it regressed with increase in cutting speed for SX9. SX9 outperformed WG300 in terms of notch wear during the tool wear process. Therefore, chemical reactions were not the primary mechanism behind notch formation.

In the present study, a 3 s short-time cutting test was conducted at a cutting speed of 310 m/min. The result showed that a notching was generated for WG300 whose notch wear VN had attained 0.2 mm, while no obvious notching was found for SX9. The observation indicated that the notching was not the result of normal wear but of brittle fracture. Accordingly, the notch formation was related to the physical and mechanical properties of the ceramic tools at its initial stage.

When cutting Inconel 718 with ceramic tools, steep temperature and stress gradients were believed to be readily generated at the depth-of-cut line of ceramic tools [16]. Thus, thermal shock resistance, which depends on fracture toughness, elastic modulus, thermal conductivity, and expansion coefficient, played an important role in the notch formation. It was reported that Si_3_N_4_-based ceramic tools possessed higher thermal shock resistance than their Al_2_O_3_-based counterparts [27,28]. It could be inferred that at the initial stage of notch formation, the excellent thermal shock resistance and high fracture toughness enabled SX9 to endure the thermal and mechanical damages at the depth-of-cut line. Although fracture toughness and thermal conductivity were enhanced with the addition of SiC whiskers, WG300–having a relatively low thermal shock resistance–failed to withstand the damages, leading to a notching in a very short time.

**Figure 9:**
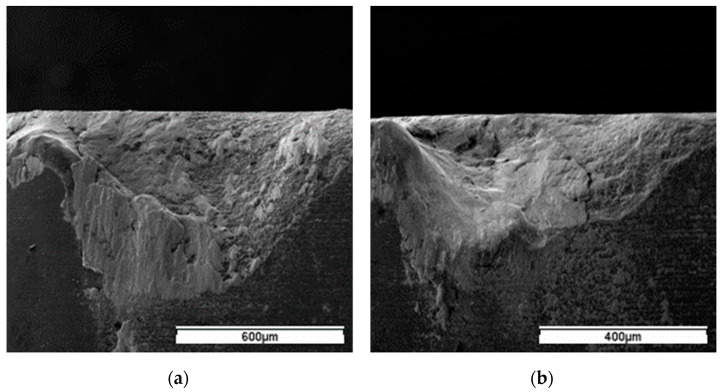
Typical notching of: (**a**) WG300 at 250 m/min; (**b**) SX9 at 200 m/min.

**Figure 10:**
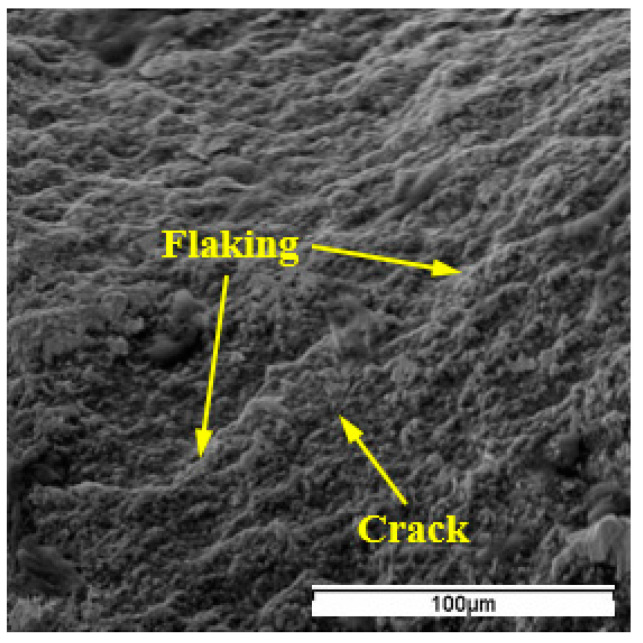
Flaking and cracks at the notching area of SX9.

**Figure 11:**
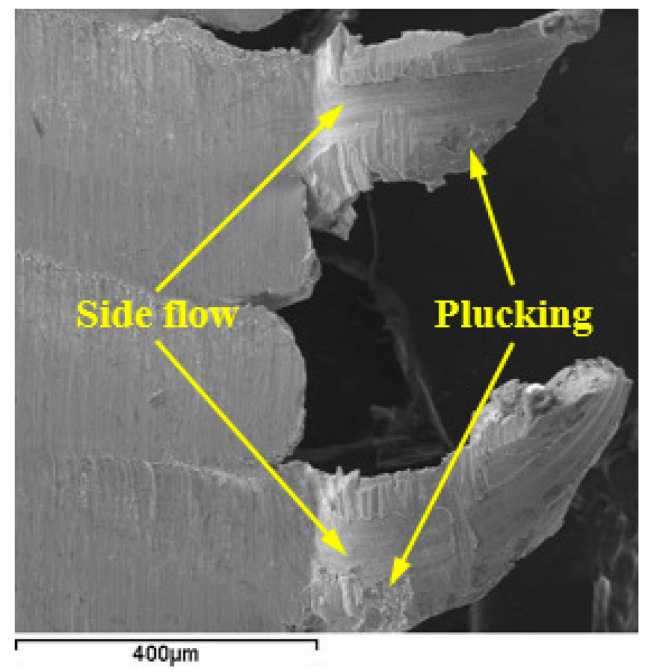
Side flow of chip at cutting speed of 200 m/min.

The notching would expand along the depth-of-cut line, and consequent notch wear VN increased with further cutting. The typical notching of WG300 and SX9 obtained at cutting speeds of 250 m/min and 200 m/min, respectively, are shown in Figure 9. The notching was characterized by adhesion of the workpiece material and an uncovered, rough appearance. At the uncovered area, cracks and traces of flaking were detected (Figure 10). The underside of the chip obtained at a cutting speed of 200 m/min showed the side flow of workpiece material occurred during the chip formation process (Figure 11). The periodic side flow of workpiece material at the depth-of-cut line resulted in the rough appearance of notching under the action of an interrupted seizure. The trace of adhesive interaction between the tool and workpiece material was also found at the extension of the chip edge, as seen in Figure 11. Therefore, at the expanding stage of notch formation, the evolution of notching was considered to be dominated by the adhesion mechanism.

**Figure 12:**
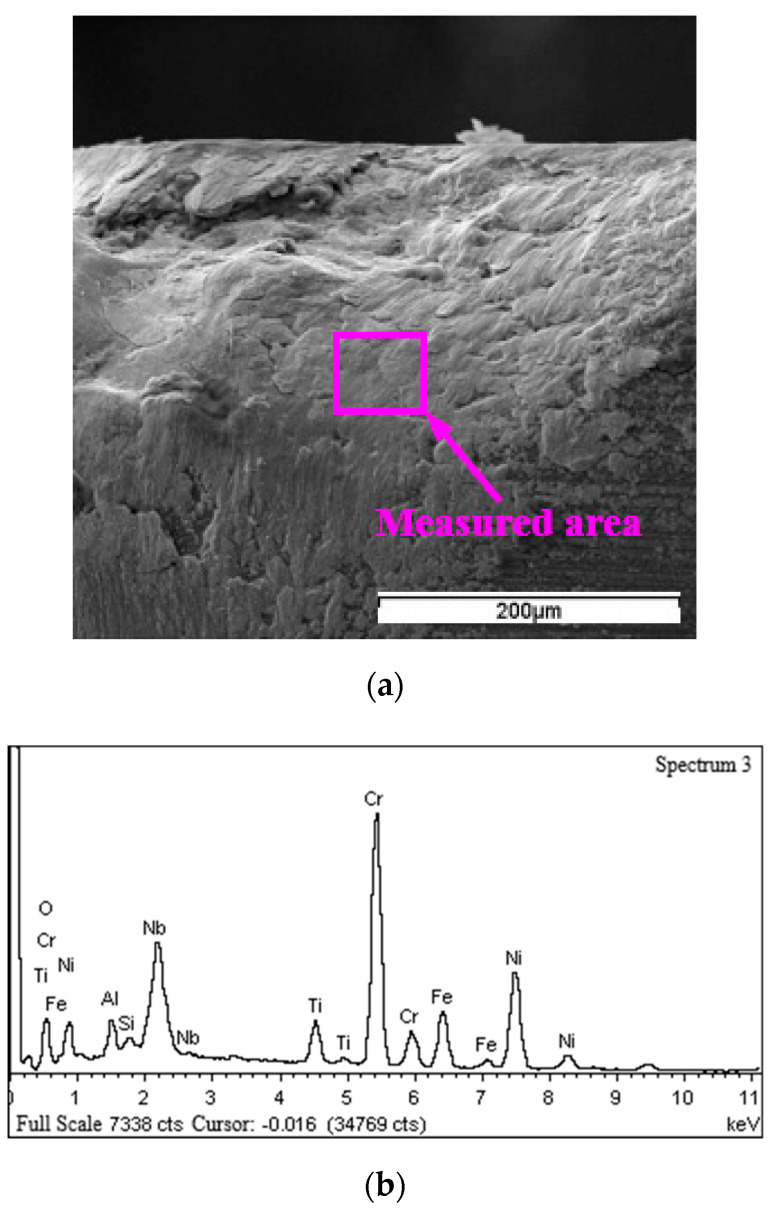
Adhered material at the notching area of SX9: (**a**) SEM micrograph; (**b**) EDS spectrum.

The EDS analysis of the adhered workpiece material at the notching was conducted to reveal its effect on the notch formation. The measured area and EDS analysis results of SX9 obtained at cutting speed of 250 m/min are shown in Figure 12. The composition of adhered material at the measured area is listed in Table 4. At the measured area, the content of element O exceeded its original content in the Sialon tool when the contents of element Si and Al at the same area were chosen as the references. Thus, the element O mainly came from the ambient air and workpiece material adhered at the notching in the form of oxides due to oxidation. The content of element Cr attained a high level compared to that in Inconel 718 alloy; one of possible oxides might be Cr_2_O_3_ [3,29]. The adhered oxides were also detected at the notching under other cutting conditions for SX9. However, such oxides were not found at the notching for WG300; the composition of adhered material was nearly identical to that of Inconel 718 alloy. The adhered oxides were perhaps covered by the outer workpiece material or had been scraped away from the notching.

Under dry cutting conditions, high cutting temperatures would be generated at the cutting zone. At the chip/tool or workpiece/tool interface, the contact was so tight that no external lubricant could penetrate it [23]. However, at the depth-of-cut line, the freshly cut workpiece material surfaces gained access to the oxygen in the air. These surfaces were chemically active and could react with the oxygen to form metallic oxides at high cutting temperatures. The relatively high contents of element O and Cr at the notching indicated that the workpiece material experienced oxidation. The oxidation of the workpiece material could reduce the affinity of the freshly cut surfaces for the tool, and thus the periodic impact of the adhered material was alleviated. A similar effect of the oxygen on the notch formation was also confirmed by the tests carried out in the presence of various active and inert atmospheres [30]. As the cutting speed increased, the high temperature oxidation of the workpiece material became severe, and its positive effect on decreasing the adhesive wear of the tool became more prominent. This conclusion explained why the notch wear was dominant at lower cutting speeds and regressed with an increase in cutting speed for ceramic tools.

### 3.6. Limitation and Future Work

The wear behaviors of different ceramic tools under high cutting speeds were evaluated in this research. The results regarding wear mechanisms and notch formation could provide reference to the design and manufacture of advanced ceramic cutting tools. The cutting parameters used in the tests are also helpful in the selection of optimal parameters for practical machining of nickel-based alloys. Although the wear mechanisms were investigated with the aid of SEM and EDS, the Ti enrichment and the related diffusion between the workpiece and cutting tools were not definitely determined. Future research should focus on the inspection and analysis of the workpiece/cutting tool interface with new techniques. In addition, the surface integrity of the machined surfaces is the other important concern during machining nickel-based alloys. The surface integrity induced by tool wear should be studied to comprehensively understand the cutting performance of ceramic tools.

## 4. Conclusions

In the present study, high-speed turning of Inconel 718 with SiC whisker-reinforced alumina and Sialon ceramic tools was conducted to investigate the wear mechanisms and notch formation. The following conclusions can be drawn:

(1) The SiC whisker-reinforced alumina WG300 and Sialon SX9 experienced distinct wear processes. The notch wear dominated the whole wear process of WG300 at low cutting speeds and the transition to flank wear occurred at the cutting speed of 370 m/min. In contrast, the notch wear of SX9 gradually decreased as the cutting speed increased, and the tool attained the wear criteria in the form of flank wear at all cutting speeds.
(2) The notching at the depth-of-cut line was the typical wear mode for WG300, while SX9 was characterized by ridges and grooves perpendicular to the cutting edge. With an increase in cutting speed, chipping of the edge and flaking of the rake face occurred for both ceramic tools. Additionally, notching at the minor cutting edge and the collapse of the tool nose were detected for WG300 at higher cutting speeds.
(3) The hard carbide particles contained in Inconel 718 together with the tool particles dropping from the tool matrix resulted in the abrasive wear of SX9 by high-frequency scraping action. In addition, tool material was plucked off from the tool matrix by periodic attachment and detachment of the adhered workpiece material, leading to the adhesive wear of the ceramic tools.
(4) The Ti−enriched belt generated at the boundary of the wear band and crater indicated the diffusion between the workpiece material and cutting tool elements. The elements Ni, Fe, and Cr diffused along the SiC whiskers or silicon nitride grain boundaries into the tool matrix, while Ti was left on the tool surface due to immediate reaction with an element from the cutting tool. The diffusion undermined the tool matrix by dissolving the SiC whiskers or silicon nitride grains.
(5) The notch formation depended on the thermal shock resistance and fracture toughness of the ceramic tools at its initial stage, while at the expanding stage, the evolution of notching was dominated by the adhesion mechanism. The high contents of element O and Cr at the notching suggested that the oxidation of the workpiece material occurred at this position. The oxidation of the workpiece material could reduce the affinity for the tool, and thus had a positive effect on decreasing the notch wear of ceramic tools.

## Figures and Tables

**Table 1:** Nominal composition of Inconel 718 (wt.%).

C	Si	Al	Mn	Co	Ti	Cu	Mo	Nb	Ni	Cr	Fe
0.052	0.26	0.56	0.22	0.5	1.08	0.1	3.03	5.03	52.15	19.26	Bal.

**Table 2:** Physical properties of Inconel 718.

Tensile Strength (MPa)	Yield Strength (MPa)	Elastic Modulus (GPa)	Hardness (HRC)	Density (g/cm^3^)	Thermal Conductivity (W/(m°C))	Melting Temperature (°C)
1430	1300	204	40	8.24	14.7	1310

**Table 3:** Cutting parameters applied in the tool wear experiments.

Cutting Parameters	WG300	SX9
Cutting speed (m/min)	190, 250, 310, 370;	150, 200, 250;
Feed rate(mm/r)	0.1	0.15
Depth of cut(mm)	1	1

**Table 4:** Composition of adhered material at the measured area.

Element	Wt. %	At %
O K	10.37	28.42
Al K	2.79	4.54
Si K	1.16	1.81
Ti K	3.98	3.64
Cr K	33.47	28.23
Fe K	10.24	8.04
Ni K	26.85	20.06
Nb L	11.14	5.26
Total	100	100

## Data Availability

Not applicable.
